# Managing Diabetes Using Mobiab: Long-Term Case Study of the Impact of a Mobile App on Self-management

**DOI:** 10.2196/36675

**Published:** 2022-04-20

**Authors:** Václav Burda, Miloš Mráz, Jakub Schneider, Daniel Novák

**Affiliations:** 1 Department of Cybernetics Faculty of Electrical Engineering Czech Technical University in Prague Prague Czech Republic; 2 Department of Diabetes Diabetes Centre Institute for Clinical and Experimental Medicine Prague Czech Republic

**Keywords:** diabetes mellitus, self-management, mobile app, case study, long-term data

## Abstract

**Background:**

This paper describes the development of a mobile app for diabetes mellitus (DM) control and self-management and presents the results of long-term usage of this system in the Czech Republic. DM is a chronic disease affecting large numbers of people worldwide, and this number is continuously increasing. There is massive potential to increase adherence to self-management of DM with the use of smartphones and digital therapeutics interventions.

**Objective:**

This study aims to describe the process of development of a mobile app, called Mobiab, for DM management and to investigate how individual features are used and how the whole system benefits its long-term users. Using at least 1 year of daily records from users, we analyzed the impact of the app on self-management of DM.

**Methods:**

We have developed a mobile app that serves as an alternative form to the classic paper-based protocol or diary. The development was based on cooperation with both clinicians and people with DM. The app consists of independent individual modules. Therefore, the user has the possibility to use only selected features that they find useful. Mobiab was available free of charge on Google Play Store from mid-2014 until 2019. No targeted recruitment was performed to attract users.

**Results:**

More than 500 users from the Czech Republic downloaded and signed up for the mobile app. Approximately 80% of the users used Mobiab for less than 1 week. The rest of the users used it for a longer time and 8 of the users produced data that were suitable for long-term analysis. Additionally, one of the 8 users provided their medical records, which were compared with the gathered data, and the improvements in their glucose levels and overall metabolic stability were consistent with the way in which the mobile app was used.

**Conclusions:**

The results of this study showed that the usability of a DM-centered self-management smartphone mobile app and server-based systems could be satisfactory and promising. Nonetheless, some better ways of motivating people with diabetes toward participation in self-management are needed. Further studies involving a larger number of participants are warranted to assess the effect on long-term diabetes management.

## Introduction

This paper describes the development of a mobile app for diabetes mellitus (DM) self-management and discusses the results of its long-term usage by selected users after 5 years. The design of the app (called Mobiab) consisted of a holistic process involving end-user requirements, expert involvement, incorporation of behavioral change theory, data security, and data privacy considerations.

DM is a chronic disease affecting large numbers of people throughout the world, and this number is continuously increasing. According to the International Diabetes Federation, there are 537 million adults worldwide has diagnosed with DM [1,2]. In the Czech Republic, in 2020, nearly 1 million people live with this disease; that is, almost 10% of the population of the country [1,3]. There are two main types of DM: type 1 DM and type 2 DM and other types such as gestational diabetes, secondary diabetes, and other forms of DM [4,5]. Type 1 DM is characterized by an absolute lack of insulin secretion from pancreatic β-cells and is responsible for approximately 5%-10% of cases [4-6]. Type 2 DM is characterized by progressive loss of insulin secretion from the pancreatic β-cells with an underlying background of insulin resistance resulting in hyperglycemia, which further leads to the development of acute and chronic complications [4,5,7]. Type 2 DM accounts for approximately 90%-95% of cases [4,5]. Self-management is essential for attaining optimal long-term glucose control, and requires careful recording of food intake, glycemic values, insulin doses, and other information. A typical part of self-management is using paper-based protocols or diaries for recording diabetes-related values [8]. This can be problematic and complicated because the person with DM has to remember or look up caloric values in different meals.

There is massive potential to increase involvement with self-management of DM using smartphones and digital therapeutics interventions. Mobile health (mHealth) applications can also reduce barriers to the availability of the health care system; for example, time constraints or limited access to care providers [9]. Smartphone apps for diabetes might have an extensive outreach, as more than 6.37 billion people in the world use smartphones [10], and approximately 0.5 billion of them already use some mobile app for diet, physical activities, and chronic disease management [11]. There are numerous mobile apps dealing with DM. For the term “diabetes,” there are more than 200 mobile apps available on the Google Play platform alone [12]. However, despite the large number of apps in this field, only a few had been evaluated in health outcome studies [12] and just 5 were associated with clinically significant improvements in hemoglobin A_1c_ (HbA_1c_) levels (Glucose Buddy, Diabeo Telesage, Blue Star, WellTang, Gather Health) [12]. These studies did not assess other parameters such as blood pressure and body weight [12]. The authors of one study identified and compared 19 mobile apps in terms of the availability of features for DM self-management [13]. Few of them have been designed on the basis of a behavioral model and have been endorsed by health care professionals. In addition, it is important to have appropriate integration without compromising user safety and privacy. The use of mobile apps can improve DM management and can contribute to education of persons with DM and motivate them to maintain healthy behavior. Several small-scale studies have shown promising results in terms of targeting blood glucose, medication intake, weight loss, and quality of life [14-17]. To our best knowledge, there is no published full report on a case study of diabetes self-management over a 5-year period.

The aims of this study are to (1) explore how long-term usage of such a system may benefit its users, (2) describe the process of development of a mobile app focused on self-management of people with DM, and (3) evaluate the demand for individual features or modules.

## Methods

### Requirement Analysis

We developed the Mobiab system within the context of OLDES (www.oldes.eu), a European Union (EU) multicenter project involving 4 companies, 2 universities, and 2 university hospitals. The OLDES project focused on developing information technology for the purposes of eHealth applications [18]. We defined the essential requirements for a system on the basis of interviews and discussions with diabetologists from the university hospital in Prague, representatives from the national Czech diabetes association, and people living with diabetes, who were recruited from an outpatient clinic at the university hospital. This approach enabled us to involve the needs of health professionals and people with DM during the design and development of the app. Additional information was gathered by searching public scientific databases using the following combinations of keywords: “mobile app,” “diabetes,” “diabetes management,” “patient adherence, empowerment,” “mobile health,” and “self-management.” Several paper-based diabetes diaries were used to define the main functionalities that were to be integrated [19].

### Architecture and System Functionalities

The Mobiab system offers an alternative to a paper-based diary—an Android mobile app and a web portal aimed at supporting DM self-management. Compared with a paper-based diary, the main benefit is the immediate feedback for inputted data in the form of graphs and basic statistics showing the user’s compliance with diet or providing self-monitoring of blood glucose levels. The Mobiab system was designed in a client-server architecture with a storage system on the server. Mobiab requires an internet connection on mobile devices. In the beginning—that is, in 2014—this approach was restricted by lower availability of internet connection [20]. However, this is no longer a problem, now that internet connection is much more widely available.

**Figure 1:**
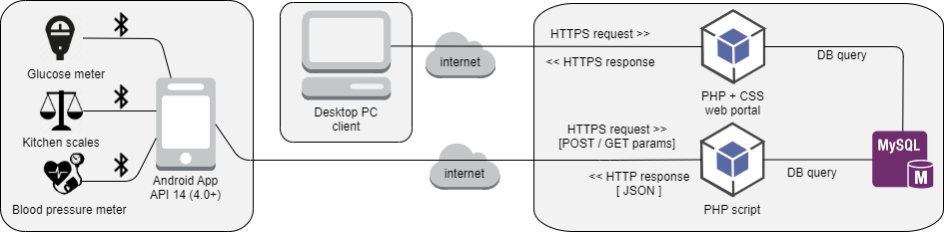
Scheme of system architecture. API: application programming interface.

The concept underlying Mobiab consists of a mobile app, data collection from medical devices, and data storage (Figure 1). All medical data are collected on a mobile phone and are stored on the server. We prepared a prototype of a Bluetooth connection to selected medical devices from ForaCare Suisse AG. The connection works fully automatically—records of measurements are downloaded and are stored without any action by the user. With users’ consent, the collected behavior data and medical data are then available on a desktop computer to selected physicians. Common security standards and privacy policies have been followed in the design and development of the Mobiab system. Communication between the smartphone or computer and the server is encrypted via the HTTPS protocol. After the app download, registration or login is initially required. The login screen requires a unique email address and password to access the app functionality. An expert group consisting of endocrinologists, health researchers, nutrition nurses, and app developers provided valuable decisions for the design and development of Mobiab.

### Description of the Mobile App and the Web Interface

**Figure 2:**
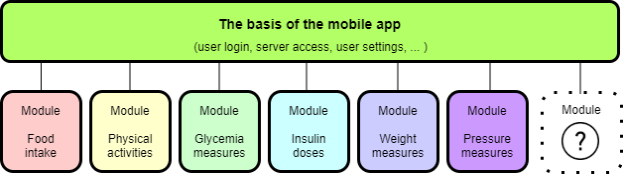
Scheme of the mobile app and individual modules.

The mobile app consists of individual modules that are independent of each other and need only the basis of the app (Figure 2). The main advantage of applying a modular approach is that other functionalities can easily be added, and particular users can select only certain modules that are suited to their needs. For example, people with type 2 DM and those who do not use insulin can turn off the insulin module. All entered values in the modules are visualized intuitively and enable the user to monitor the changes continuously. A description of the modules with their main features is provided below.

#### Food Intake and Physical Activities

Food intake is the most complex module and provides the functionality for recording food that is consumed. This module now contains a food database with more than 9000 Czech food items. The database has gradually been expanded and checked for data accuracy by other users. There are several approaches to food consumption logging:

- Search in the whole database
- Search in favorite items
- Browse all food items and filter by categories
- Browse user’s meals or simply take a photo of the food.

**Figure 3:**
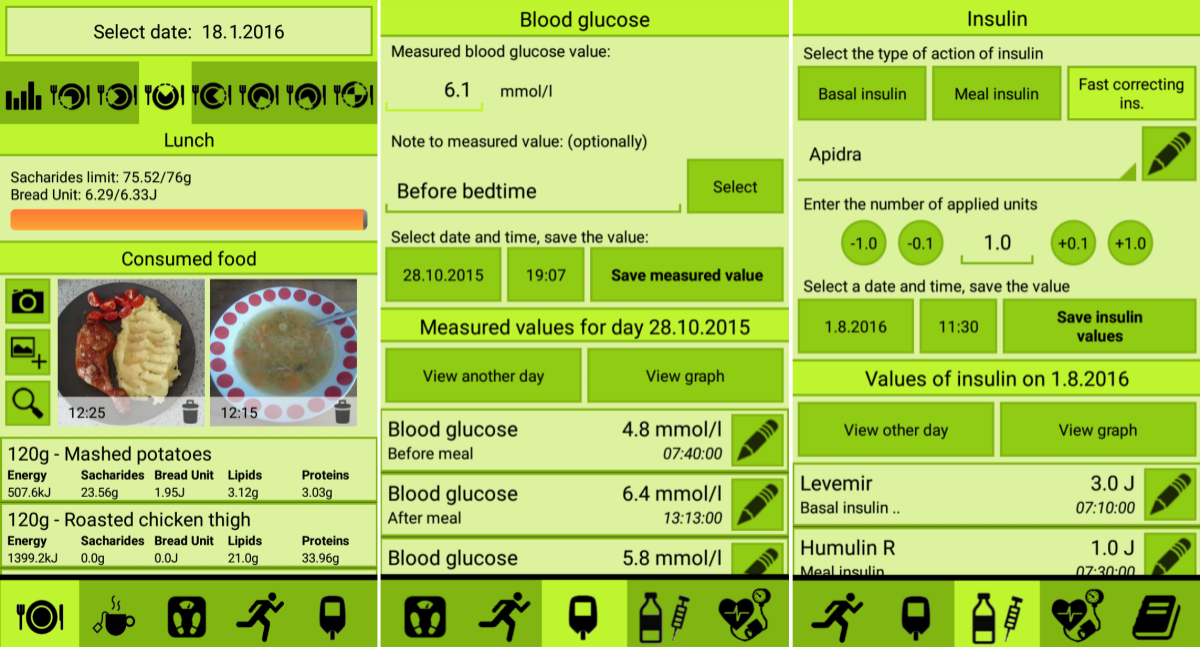
Screenshots of the mobile app: food intake, glycemia monitoring, and insulin doses.

The user enters the amount of food after searching for the specific food item. The time stamp for the consumption and the food category is predefined by the current time; however, this can be changed by the user. To enable the user to change their mind, the description of the nutrition, and the size of the portion (in grams), and the carbohydrates (in grams) are displayed before the final dialog is saved. The changes in values are facilitated by an intuitive visualization of all measured medical data (Figure 3).

The physical activities module was designed similarly to the food intake module: the database contains more than 400 activities that can be browsed by categories or searched by name. It is necessary to select one activity and to enter the duration of the activity for logging. The caloric expenditure is computed with the user’s weight and the duration of the activity. Owing to this approach, the computed caloric expenditure may not always match the real expenditure and should be considered solely a guide.

#### Glycemic Monitoring and Insulin Dosage

The Glycemic Monitoring module has a simple design for easy usage. It has an input part for entering values; for example, glucose levels, the date and time of measurement, and notes. The second part of the module is an overview of the values for the selected day, or a graph for the selected time range (Figure 3). The insulin applications module is more complex than the previous modules. As shown (Figure 3), the user first chooses from among 3 types of insulin (basal, prandial, and fast correcting), then selects a specific brand name of insulin (user editable), the number of applied units, and the date and time of application. The overview section is the same as in the Glycaemia Monitoring module.

### Data

Data were collected through Mobiab over a period of 5 years (from January 2016), although Mobiab had been available on Google Play Store from mid-2014 only until 2019. No advertisement was used to recruit users, they found the mobile app in an organic reach. Over this period, over 500 users from the Czech Republic, who used the app for different lengths of time. Approximately 200 users did not report any DM, approximately 150 users reported type 1 DM, and approximately 175 users reported type 2 DM. Approximately 80% of the users used the mobile app for less than one week. The remaining 20% of the users used Mobiab for a longer time with a decreasing usage trend as it was also noted previously [21]. However, only those satisfying at least one of the following conditions were selected for the analysis:

1. At least 3600 records of food intakes
2. At least 360 records of glycaemia measurements
3. At least 360 records of insulin doses
4. At least 1080 records of physical activities
5. At least 360 records of weight measurements
6. At least 360 records of pressure measurements.

Meeting one of these conditions was considered to provide evidence of long-term usage. Details about users (Table 1) and the number of records and records per day (Table 2) are shown in tables.

**Table 1:** Basic users’ statistics.

User ID	Sex	Birth year	Height (cm)	Diabetes mellitus type	Active days, n
ID 1141	Male	1962	173	Type 2	1749
ID 1196	Male	1960	178	Type 2	1261
ID 1224	Female	1976	162	Type 1	1623
ID 1289	Female	1941	162	No diabetes	1626
ID 1412	Female	1976	162	Type 2	96
ID 1432	Male	1958	175	Type 1	804
ID 1545	Male	1967	188	Type 2	881
ID 1558	Male	1967	170	Type 2	247

**Table 2:** Number of records and daily averages.

Patient ID	Food entries, n (daily mean)	Glycemic measures, n (daily mean)	Insulin doses, n (daily mean)	Physical activities, n (daily mean)	Weight measures, n (daily mean)	Pressure measures, n (daily mean)
ID 1141	34425 (19.67)	—^a^	—	13,515 (7.73)	1690 (0.97)	1478 (0.85)
ID 1196	—	1164 (0.92)	—	—	—	—
ID 1224	9470 (5.83)	1932 (1.19)	2166 (1.33)	4562 (2.81)	—	449 (0.33)
ID 1289	15,729 (9.67)	—	—	—	—	—
ID 1412	—	466 (4.85)	—	—	—	—
ID 1432	3757 (5.86)	799 (0.99)	1199 (1.53)	2982 (4.18)	—	—
ID 1545	—	857 (0.97)	697 (0.79)	—	—	859 (0.98)
ID 1558	—	538 (2.18)	—	—	—	—

^a^—: not available.

In total, 8 users (5 male, 3 female) fulfilled the inclusion criteria for long-term analysis, 5 of whom stated that they had type 2 DM, 2 had type 1 DM, and 1 was without DM. The average age of all users was approximately 57 years. All 8 users were invited to provide medical records, but only one user (ID 1141) was willing to share them. We were particularly interested in the development of the following clinical parameters during use of the app: hemoglobin A_1c_ (HbA_1c_), glycemia, triglycerides, and cholesterol (total, low-density lipoprotein, and high-density lipoprotein cholesterol). The summary of records for the whole 7 years of the user who provided medical records are presented in Table 3. The frequency of the laboratory’s clinical parameters is sufficient to draw a conclusion about the progress in DM treatment [22]. In addition to these medical records, user ID 1141 provided personal health state remarks that are presented in the case study results.

The analysis of the data had to use two approaches owing to missing user medical records: the first approach is an analysis of usage of the application, including any beneficial trends for DM management, and the second approach is to make a direct comparison between the medical records and the entered values and trends of the user ID 1141.

**Table 3:** Selected medical records of user ID 1141.

Date	Hemoglobin A_1c_ levels (mmol/mol)	Glycemia (blood glucose measured in terms of mmol/L)	Total cholesterol (mmol/L)	Low-density lipoprotein cholesterol (mmol/L)	High-density lipoprotein cholesterol (mmol/L)	Triglycerides (mmol/L)
January 1, 2014	—^a^	4.6	4.3	2.82	1.15	1.07
April 17, 2016	—	18.17	—	—	—	—
April 26, 2016	90	7.9	4.17	2.62	1.4	0.85
July 21, 2016	36	4.7	—	—	—	—
November 11, 2016	29	5	4.58	2.39	1.49	0.67
March 6, 2017	32	4.8	3.81	1.98	1.66	0.53
July 17, 2017	34	4.5	4.05	—	—	0.81
April 23, 2018	35	5.3	3.73	2.08	1.48	0.51
September 17, 2018	34	5	4.11	2.52	1.55	0.73
February 4, 2019	—	4.9	3.96	2.54	1.18	0.93
June 17, 2019	35	4.9	3.66	2.03	1.46	0.6
November 4, 2019	35	5.1	4.26	2.57	1.39	0.99
March 19, 2020	36	4.7	3.3	1.65	1.44	0.5
July 13, 2020	35	5.1	3.78	2.09	1.44	0.65
November 13, 2020	36	5.2	3.77	2.01	1.62	0.64
March 22, 2021	35	5.4	3.71	1.98	1.53	0.78

^a^—:not available.

### Ethical Considerations

Ethics approval from the ethics committee of our university was not required for this study. All users agreed to use anonymized data for purposes of research and data analysis during sign-up process, which is required for the app usage.

## Results

### Analysis of Usage

At first, we analyzed the long-term food intake. Users ID 1141 and ID 1289 recorded their food intake regularly. They were strictly taking their diet plan and followed energy and sugar intake limits. User ID 1141 still uses the mobile app, and his performance is described in detail in the following section. Two other users, ID 1224 and ID 1432, enter data irregularly every few days.

**Figure 4:**
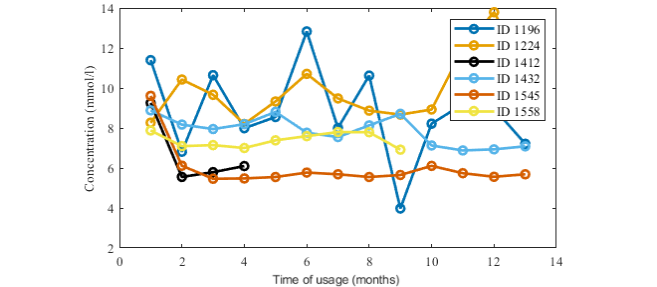
Records of blood sugar in the first year of app use.

Nevertheless, ID 1224 used the app for over 4 years, and ID 1432 used it for 2 years. Interestingly, both of these users has type 1 DM, and they used the app much more regularly for entering glycemic values and insulin dosage than for food intake recording. The glycemic records (Figure 4) show a slight decrease in blood glucose levels after a few months of usage of Mobiab. A more important fact for our study’s purposes is that users ID 1224 and ID 1289 carried out long-term recordings and were engaged for more than 2 years, and users ID 1432 and ID 1545 were engaged for more than 4 years. Additionally, the other users, ID 1412 and ID 1558, were involved with Mobiab for a shorter time, for 3 months and 8 months, respectively, but during that time they regularly included several measurements per day.

### Case Study of App Use With Type 2 DM

**Figure 5:**
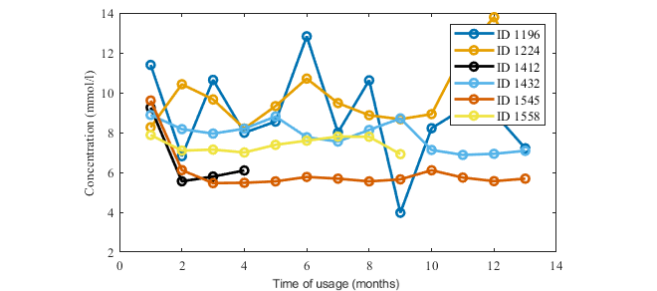
Weight and waist circumference records for the entire period of app usage.

**Figure 6:**
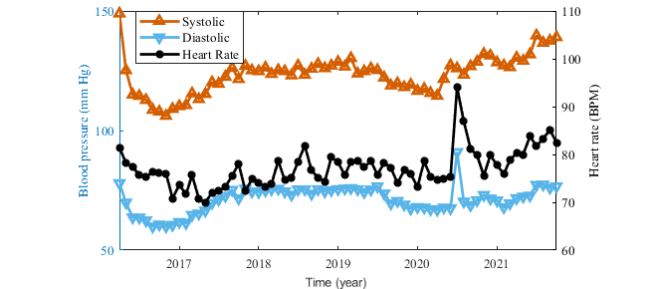
Blood pressure records for the entire period of app usage.

**Figure 7:**
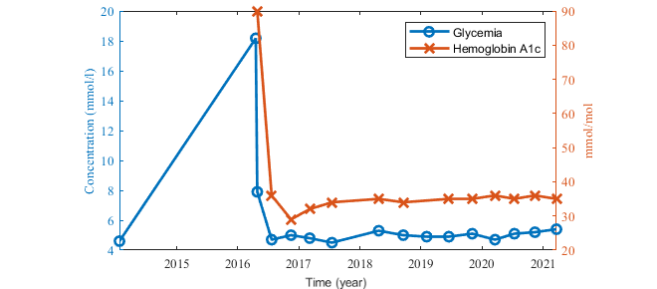
Medical records for hemoglobin A_1c_ and glycemia.

**Figure 8:**
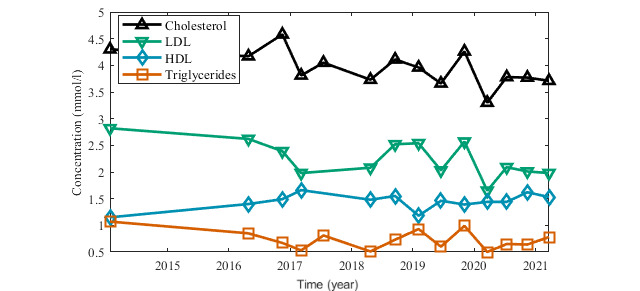
Medical records for cholesterol and triglycerides. HDL: high-density lipoprotein, LDL: low-density lipoprotein.

User ID 1141 (male, 60 years old, type 2 DM) was selected for the case study because he was willing to share his medical records and other information about his health and lifestyle. This person had been diagnosed as having type 2 DM randomly during an emergency examination on April 17, 2016. Before that, he had already been treated for high blood pressure and for hyperlipidemia. After the diagnosis of DM, he has been treated with medication (Glucophage XR, 500 mg) and he had been looking for some supporting mobile app. He started dieting and the records show that he has followed the diet constantly for the whole time that he has used the app. In total, he has entered over 34,000 food records. Positive results were soon obtained. With regular exercise (stationary exercise bike and walking) he reduced his weight from 127 kg to 84 kg, and his waist circumference decreased from 141 cm to 107 cm within 1 year. In the last 3 years, these values have increased moderately, as of March 2021, his weight was 101 kg because he has not been able to exercise intensely owing to joint pain and he stopped entering new waist circumference values (Figure 5). His blood pressure and cholesterol levels have also improved and then stabilized (Figure 6). All these results are in accordance with his medical records (Figure 7). Unfortunately, the person does not self-monitor blood glucose, and only periodical medical records of his glycemic levels are available (Figure 8). Based on the usage quality questionnaire and a semistructured interview, he was very satisfied with the mobile app and appreciated how easy the app was to use. As of this writing, he is still using Mobiab, and he will complete 8 years of usage in April 2022.

## Discussion

### Principal Findings

The main goal of the Mobiab system is exploring benefits of long-term usage of technology for DM self-management. The system simplifies manual entering and documenting of measured values associated with treatment monitoring and self-management of DM and provides a user-friendly summary of their self-management efforts. The Mobiab system contributes to the user’s education and a better understanding of the disease by providing continuous recordings of all essential data, including food intake, caloric expenditure, blood glucose levels, insulin dosage, body weight, and blood pressure. In addition, we might argue that the Mobiab system contributes to long-term outcomes of DM management, as demonstrated in several use cases. Several studies have suggested the usefulness of electronic self-management systems in managing DM [23]. For example, smartphone apps have been shown to improve glycemic control, specifically in younger people with DM [24]. Another randomized controlled trial showed that DM intervention using smartphones led to improved clinical outcomes [25]. The US Food and Drug Administration has now approved several (BlueStar) mobile apps for DM management [25], and the new German Digital Health Applications (in German: “Digitale Gesundheitsanwendungen”) scheme has also been approved [26]. These data confirm an increasing trend to introduce digital therapeutics intervention into daily clinical practice [27]. A further benefit of smartphone apps is that anonymized data can be collected from a larger population.

The collection of medical data using Mobiab was beneficial to users with both diabetes types. Previously, it had been necessary for people with DM to record medical values manually in a diabetes diary. Using Mobiab, user ID 1141 has already been able to record his food consumption, exercises, weight changes, and blood pressure continuously for 1749 days. In addition, the user achieved positive changes in blood glucose levels (Figure 7) and weight control (Figure 5) within a concise time. Although we cannot quantify the exact contribution of the Mobiab app to these improvements, the benefits for user ID 1141 have been considerable. A positive impact of the assistance of the mobile app on diet and blood glucose levels were also confirmed in a study focusing on mySugr app benefits [28].

Some systems applied training participants ranging from telephone [29] to face-to-face support [30]. The design of the app followed the user-centered design and the final design was also commented on by the expert group. At the end, no personal training was offered to participants, since we assumed that the app is easy and intuitive to use. However, the onboarding procedure explaining the main app functionalities started after installing and launching the app.

Only a few technology-related issues were reported. The main comments stemmed from the use of the app without an internet connection, mainly at the beginning of the app launch. While there was considerable effort to ensure complete app functionality without the internet connection by caching all parameters as in the case of earlier systems [31]; after several updates, it was decided to remove this feature. This is in line with most of the current solutions based on cloud architecture, which requires a stable connection to guarantee smooth operation [32]. Further, no similar studies analyzed the number of calls that participants or clinicians made for technological support [33].

The Mobiab data set is highly variable in terms of the usage of the modules. Not every user used the same set of modules (Table 1). This is a limiting factor for a complex analysis of the health impacts. However, this variability of usage of the modules should not be classified as an app issue because it only indicates the well-known highly heterogeneous needs of people with diabetes [34]. The hypothesis that engaged participants used more modules reflecting their higher discipline was not confirmed in our study. Nevertheless, the Mobiab developers will continue to make modules more attractive to users and convince people with DM that it would also be beneficial to use a broader range of modules; for example, to provide overviews of complex data and explain the impacts on their health. We believe that a broad selection of modules is advantageous for people with DM, thus contributing to personalized DM self-management, increasing the participants' engagement and long-term outcomes [35-37]. Furthermore, several studies discussed the usefulness of using the Chronic Care Model to improve clinical and behavioral outcomes applying eHealth technology. Consequently, we have identified several improvements that might reduce the burden of the disease and increase engagement by expanding the modular architecture [38-40]. Combination of general and tailored educational content might help cope with medical jargon and misleading information from different sources. In addition, tracking mood on a daily or weekly basis might be important to provide insight for better glycemic control and to prevent depression and diabetes distress [41,42].

However, there is a concern about placing too much confidence in managing DM using mHealth apps [29,30,43]. These pilot studies have pointed out that some people with type 2 DM do not believe in the benefits of these apps resulting in a low level of usage [30,44-46]. When discussing self-management of diabetes with the use of a mobile app, several research papers have emphasized the need for education, peer support, interactive content, blood glucose monitoring, dietary tracking, and realistic goal setting [22,47-50]. Another important concept for increasing the efficacy of interventions is the establishment of a 2-way communication between the patient and care team [51]. We supported this type of communication by developing a stand-alone web-based clinical portal for physicians.

However, the long-term usage of apps developed for managing DM using self-management tools remains low [44]. Our own experience suggests that our app can achieve good outcomes, but it is not straightforward enough to motivate people with diabetes to self-manage their condition consistently in the long term. Long-term engagement with mHealth systems does not necessarily require daily interaction; routine DM management could lead to the reduction of using the technology [21].

Most of the studies referenced in this paper were single-center pilots validating short-term results of the examined mobile apps. Undoubtedly, more clinical trials with extended follow-up periods are needed to evaluate the long-term effect of diabetes-related mobile apps on glucose management and quality of life, and sustainability of self-management using the mHealth ecosystem [52]. A clinical study for validating the impact of the Mobiab system on self-management behavior and for exploring the usability of the system is currently under development.

### Strengths and Limitations

A major strength of this study is the involvement of 5 persons with type 2 DM, 2 persons with type 1 DM, and 1 person without DM, each of whom could use the system for a long time and enter a significant amount of data. However, the small number of participants is a limitation of our study. A very small set of users is insufficient to thoroughly test and validate the self-management compliance of the Mobiab system. In addition, even this small number of participants did not use all the modules that the system provides.

Another limitation is the integration of only one glucometer. We implemented seamless glucose data transfer using a specific glucose meter (Fora Diamond MINI) and blood pressure monitor (Fora Active P30 Plus). Technical documentation and cooperation with manufacturers would be needed to connect other devices.

A further limitation is the web-based portal for physicians. A total of 5 clinicians in our expert advisory group indicated that clinicians already use some commercial software (eg, Medtronic CareLink), and that the use of different software is an unnecessary complication. The solution would be to have a communication interface to connect the mobile app to an already established system. Data integration with existing hospital information systems was not implemented as a part of our work, because we had no specification of the communication interface. However, this integration activity remains open for future work, when new versions of the hospital system are incorporated with application programming interface functionality.

### Conclusions

The results of this study have shown that the usability of a smartphone app and server-based systems are potentially satisfactory and promising. The collection of long-term data on diabetes and overall metabolic management can be supported by a modular app such as Mobiab. Our system, based on the needs and requirements of its intended users, has attempted to maximize the potential to enhance self-management and increase user adherence. In this study, 8 users evaluated app functionality in long-term monitoring. A case study has presented and analyzed the particularly successful involvement with the system. However, we cannot yet claim that the Mobiab app provides people with diabetes a well-utilized tool for their self-management to help prevent complications. An assessment of the effectiveness of the app in improving self-management over time requires further studies involving a larger number of participants. Some redesign of the mobile app will probably be required owing to continuous changes in the development of mobile apps. However, the principles of the modules and functions work well and will likely be preserved.

## Abbreviations

DM: diabetes mellitus
EU: European Union
mHealth: mobile health
